# Predictors of Age of Diagnosis and Survival of Alzheimer’s Disease in Down Syndrome

**DOI:** 10.3233/JAD-170624

**Published:** 2017-12-19

**Authors:** Amanda Sinai, Claire Mokrysz, Jane Bernal, Ingrid Bohnen, Simon Bonell, Ken Courtenay, Karen Dodd, Dina Gazizova, Angela Hassiotis, Richard Hillier, Judith McBrien, Jane McCarthy, Kamalika Mukherji, Asim Naeem, Natalia Perez-Achiaga, Khadija Rantell, Vijaya Sharma, David Thomas, Zuzana Walker, Sarah Whitham, Andre Strydom

**Affiliations:** a Division of Psychiatry, University College London, London, UK; b Sheba Medical Center, Tel Hashomer, Israel; c Cornwall Partnership Foundation NHS Trust, UK; d Westminster Learning Disability Partnership, Central and North West London NHS Foundation Trust, London, UK; e Livewell SouthWest (CIC), Plymouth, UK (data from South London and Maudsley NHS Foundation Trust, Maudsley Hospital, London, UK); f Plymouth University Peninsula School of Medicine and Dentistry, Plymouth, UK; g Barnet Enfield and Haringey Mental Health NHS Trust, UK; h Surrey and Borders Partnership NHS Foundation Trust, Epsom, UK; i Enfield Integrated Learning Disabilities Service, Enfield, UK; j Camden and Islington NHS Foundation Trust, London, UK; k Plymouth Teaching Primary Care Trust (now known as Livewell Southwest CIC), UK; l Institute of Psychiatry, Psychology and Neuroscience, King’s College London, London, UK; m Hertfordshire Partnership University NHS Foundation Trust, UK; n Sutton MHLD Team, SW London and St George’s Mental Health NHS Trust, Surrey, UK; o St George’s University of London, UK; p Royal Borough of Kensington and Chelsea Learning Disabilities Service, London, UK; q Institute of Neurology, Queen Square, Education Unit, London, UK; r Department of Community Mental Health, RAF Marham, Norfolk, UK (data from East London NHS Foundation Trust, UK); s Essex Partnership University NHS Foundation Trust, UK; t The LonDownS Consortium

**Keywords:** Alzheimer’s disease, dementia, Down syndrome, mental retardation, survival

## Abstract

**Background:**

People with Down syndrome (DS) are an ultra-high risk population for Alzheimer’s disease (AD). Understanding the factors associated with age of onset and survival in this population could highlight factors associated with modulation of the amyloid cascade.

**Objective:**

This study aimed to establish the typical age at diagnosis and survival associated with AD in DS and the risk factors associated with these.

**Methods:**

Data was obtained from the Aging with Down Syndrome and Intellectual Disabilities (ADSID) research database, consisting of data extracted from clinical records of patients seen by Community Intellectual Disability Services (CIDS) in England. Survival times when considering different risk factors were calculated.

**Results:**

The mean age of diagnosis was 55.80 years, SD 6.29. Median survival time after diagnosis was 3.78 years, and median age at death was approximately 60 years. Survival time was associated with age of diagnosis, severity of intellectual disability, living status, anti-dementia medication status, and history of epilepsy. Age at diagnosis and treatment status remained predictive of survival time following adjustment.

**Conclusion:**

This study provides the best estimate of survival in dementia within the DS population to date, and is in keeping with previous estimates from smaller studies in the DS population. This study provides important estimates and insights into possible predictors of survival and age of diagnosis of AD in adults with DS, which will inform selection of participants for treatment trials in the future.

## INTRODUCTION

Down syndrome (DS) is primarily due to trisomy of chromosome 21, which includes the amyloid precursor protein (*APP*) gene. Triplication of this gene is associated with overproduction of amyloid-*β* with amyloid deposits in the brains of virtually all adults with DS and trisomy 21 is therefore a genetic form of Alzheimer’s disease (AD) alongside mutations in *APP* which is associated with familial AD [1]. Life expectancy for people with DS has improved dramatically over past decades, from just 12 years in 1942 to 60 years in 2003 [2], revealing early onset dementia as a major clinical concern in this population. However, reported prevalence of clinical dementia varies despite the neuropathological hallmarks of AD being present in the brains of all adults with DS by age 35 [3]. Dementia prevalence has been estimated to increase from 9% between the ages of 45– 49 years; 18% between ages 50– 54 years; and 32% between ages 55– 59 years, reflecting a doubling of prevalence every 5 years [4], while longitudinal studies estimated cumulative risk to be 90% by age 65 [5]. However, these studies have included relatively small numbers of individuals with DS and dementia, and further information on the characteristics and prognosis of individuals with DS and dementia is required.

Clinical factors that predict age of onset of sporadic AD in the general population include female sex [6], lifestyle factors as indicated by cardiovascular risk factors [7] and midlife adiposity [8], physical and mental health co-morbidities, and psychosocial factors including lower educational and occupational level as well as age at retirement [9, 10]. After diagnosis, women may have a faster rate of cognitive and functional decline [6, 7] and higher educational attainment have been shown to be associated with more rapid decline [7, 11]. Age at diagnosis may also affect rate of decline [11]. With regards to genetic factors, *APOE* genotype is known to affect age at onset [12] and in individuals with familial AD, the mutation-type appears to have a strong effect on age of onset [13].

Factors associated with the large variation in age of clinical presentation of AD and subsequent rate of decline in DS remain unclear. Given that this is an ultra-high risk population with AD without significant cardiovascular risk factors, it is important to understand the factors associated with both age of onset and survival in this population as it could highlight factors associated with modulation of the amyloid cascade.

This study aimed to address these shortcomings by investigating the natural history of dementia in a cohort of individuals with DS who have been diagnosed with dementia in England, UK. This dataset allows longitudinal analysis of the course of dementia in a large clinically representative sample of adults with DS including participants from both institutional and community living backgrounds, and individuals across the range of severity of intellectual disability (ID). We aimed to establish the typical age at diagnosis and survival associated with AD in DS and the risk factors associated with these. We were particularly interested in whether age at diagnosis and survival were influenced to the same extent as in sporadic AD (SAD) by sex/gender and co-morbid health conditions.

## MATERIALS AND METHODS

### Sample

The Aging with Down Syndrome and Intellectual Disabilities (ADSID) research database consists of data extracted from clinical records of patients referred to Community Intellectual Disability Services (CIDS) for memory screening or dementia assessments for adults with ID in England. Data was extracted from clinical records using pseudonymized and standardized record forms, allowing for longitudinal tracking of participants’ assessments from first screening. Data extraction varied according to site, in some cases clinicians anonymized and recorded data, in other cases, researchers visited the site and anonymized and recorded data on site. Participating areas included services with sufficiently detailed assessments available from London and Southern England. All adults with DS who had received baseline screening or an assessment for dementia by CIDS were eligible for inclusion in the database, providing a clinically representative sample of adults with DS across the range of ID and residing in a variety of settings. For the present analysis, we selected all cases with both DS and a confirmed diagnosis of dementia at their most recent assessment. Cases where DS and/or current dementia status was recorded as uncertain, and when date of diagnosis was not recorded, were excluded.

### Ethical review and approvals

Approval was given by the central NHS Research Ethics Service as well as the National Information Governance Board (NIGB) which allowed us to collect pseudonymized retrospective data without requiring individual consent. This was necessary in order to use data relating to patients who haddied.

### Variables of interest

Variables extracted for this analysis were age and date at diagnosis, date of death or date of last assessment if patient was alive, sex, severity of ID, health co-morbidities (epilepsy, history of depression and/or anxiety, thyroid disorder, and sensory impairment), living situation, region, and anti-dementia medication status.

### Definition of dementia

Dementia was diagnosed after comprehensive assessment by clinicians based in participating CIDSs, with experience in assessing DS individuals for dementia. Diagnostic assessments typically included a detailed history of cognitive symptoms (in most areas supplemented by validated tools such as the Dementia questionnaire for People with ID) [14], medical history, routine health check followed by investigations as required to exclude untreated physical health problems, psychiatric evaluation to exclude mental illness if indicated, and neuropsychological testing although specific tests varied between services.

In instances when a possible dementia diagnosis was recorded but then removed before being re-diagnosed at a later date, date of diagnosis was recorded as the date from which a consistent dementia diagnosis was given at all subsequent assessments.

### Other definitions

*Severity of Intellectual Disability* was defined according to descriptions in the ICD-10 classification; we used three categories (mild, moderate, severe to profound). Categorization was determined by IQ if a standardized score at first assessment was available or by British Picture Vocabulary Scale (BPVS) [15] at first assessment if IQ was not available. BPVS raw scores were converted to age equivalents. BPVS age >6 years old was classified as mild ID, age 3– 6 years as moderate ID, and age <3 years as severe to profound. If neither IQ nor BPVS scores were available, severity was as recorded in clinicalnotes.

*Health co-morbidities*: Epilepsy was defined according to presence or not of pre-existing clinically diagnosed epilepsy recorded at first assessment to capture those with epilepsy morbidity prior to dementia-related seizure onset.

*History of depression and/or anxiety* was based on any medical history of clinically diagnosed depression and/or anxiety at first assessment.

*Thyroid disorder* was defined as a recorded diagnosis of thyroid disorder at *any* assessment. If specific thyroid disorder information was not provided but medication history was available, common thyroid medications (thyroxine, levothyroxine, and carbimazole) were taken as confirmation of thyroid disorder.

*Sensory impairment* was defined according to visual and/or hearing impairment being recorded in the clinical notes at first assessment.

*Living status* denotes whether a patient was living with family or away from family at first assessment. While living status does change and dementia in the DS population is associated with relocation as care needs change, this variable should capture living situation prior to and during the time of dementia onset.

*Anti-dementia drug treatment*: treatment status has been defined according to whether the patient had taken any relevant drugs (donepezil, galantamine, rivastigamine, and memantine) at any recorded time point, creating a binary (yes/no) composite covariate.

*Age of onset* was defined as time in years between date of diagnosis and date of birth.

*Survival time* was defined as the time from the initial date of diagnosis of dementia until the date of death or last follow-up.

### Analysis

All participants alive at time of data collection were censored at the date of their last assessment. If participants had no further assessments after diagnosis, and no date of death was known, duration of disease could not be calculated and these participants were necessarily excluded from analyses of survival.

*T*-tests or ANOVA were used to assess the relationship between age at diagnosis and patients’ clinical and demographic characteristics. We used the Kaplan-Meier method to estimate the median survival [16]. Univariable Cox regression models were fitted for each predictor. The proportional hazards assumption required by the Cox model [17] was investigated using Schoenfeld residuals [18]. In addition, for numerical predictors the assumption of linear relationship was also checked.

Multivariable analyses were used to estimate the combined effect of the predictors on both age at diagnosis and survival models. We used the enter procedure to adjust for confounding, using a 20% significance level.

Data analyses were carried out in SPSS v 22.

## RESULTS

The sites included in this analysis included data from 839 individuals with DS of whom 254 (30.3%) had dementia and 251 (55.4% male) participants were eligible for this study with a recorded date of diagnosis. Follow-up data post-diagnosis required for survival analysis was available for 194 (77.3%) participants. Comparisons between those with and without follow-up data revealed no differences in demographics (sex, living situation or age at diagnosis) but there were significant regional differences in follow-up rates. Region was therefore used as fixed effect in subsequent survival analyses. Table 1 displays the characteristics of eligible participants and missing data for each variable.

**Table 1:** Description of demographic and clinical characteristics

	All *n* (%) 251
Total
Age of Onset in Years
Mean (SD)	55.81 (6.29)
Sex
Female	112 (44.6)
Male	139 (55.4)
Level of Intellectual Disability
Mild	28 (11.2)
Moderate	59 (23.5)
Severe	48 (19.1)
Missing	116 (46.2)
Living
With family	44 (17.5)
Other	188 (74.9)
Missing	19 (7.6)
Epilepsy (Longstanding)
Present	46 (18.3)
Absent	161 (64.1)
Missing	44 (17.5)
History of Depression
Present	26 (10.4)
Absent	146 (58.2)
Missing	77 (30.7%)
Thyroid Disorder
Present	96 (38.2)
Absent	92 (36.7)
Missing	63 (25.1)
Sensory Impairment
Present	134 (53.4)
Absent	58 (23.1)
Missing	59 (23.5)
AD Medication (acetylcholinesterase
inhibitors or memantine)
Prescribed	72 (28.7)
Not prescribed	162 (64.5)
Missing	17 (6.8)

### Age of dementia diagnosis and predictors

**Fig.1:**
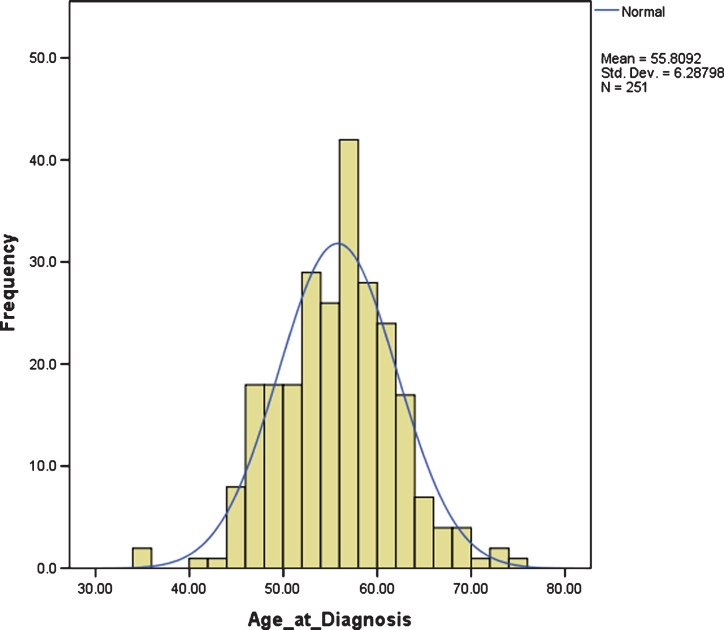
Distribution of age at dementia diagnosis.

The mean age of diagnosis was 55.80 years, SD 6.29. There was large variation in age at diagnosis, ranging from 35.46 years to 74.46 years, but it was normally distributed, with an interquartile range between 51.67 years and 59.79 years; i.e., approximately 50% of individuals with DS and dementia were diagnosed in their 6th decade (Fig. 1).

Table 2 displays differences in age at diagnosis by clinical and demographic variables. Men tended to be diagnosed slightly later than women (56.59 years, SD 6.65 versus 54.84 years, SD 5.68; *p* = 0.029), and other variables associated with age at diagnosis included living situation and sensory impairment. We therefore conducted a multivariate regression analysis (*n* = 187) using the variables associated with significant differences in age at diagnosis to adjust for confounding. The corresponding estimates and 95% confidence intervals (CI) for gender, sensory impairment and living situation were: – 0.64 (– 2.37– 1.08; *p* = 0.463), – 1.86 (– 3.75– 0.03, *p* = 0.054), and 3.24 (1.01– 5.46, *p* = 0.005), respectively (*R*^2^ = 0.078, *F* = 5.182, *p* = 0.002). Living situation remained the only significant predictor of age at diagnosis, though presence of sensory impairment showed a trend toward statisticalsignificance.

**Table 2:** Comparison of age at diagnosis by demographic and clinical characteristics

	Age at diagnosis Mean (SD)	Difference 95% CI	*p*– value
Sex			0.029^*^
Female	54.84 (5.68)
Male	56.59 (6.65)	1.74 (– 0.18 to 3.31)
Level of Intellectual Disability			0.171
Mild	55.05 (7.65)
Moderate	54.30 (5.01)
Severe	56.61 (6.89)
Living Situation			<0.001^**^
With family	51.93 (6.04)	– 4.608 (– 6.61 to – 2.60
Other settings	56.54 (6.08)	reference
Epilepsy			0.649
No	55.37 (6.51)	reference
Yes	55.86 (6.21)	0.49 (– 1.63 to 2.62)
AD Medication			0.099
No	56.21 (6.65)	reference
Yes	54.74 (6.12)	– 1.49 (– 3.23 to 0.28)
History of Depression			0.618
No	55.80 (6.47)	reference
Yes	55.11 (6.66)	– 0.69 (– 3.42 to 2.04)
Thyroid Disorder (*n* = 188)			0.425
No	55.25 (6.84)	reference
Yes	55.96 (5.11)	– 0.71 (– 2.45 to 1.04)
Sensory Impairment (*n* = 192)			0.015^**^
No	54.18 (7.00)	reference
Yes	56.74 (5.49)	2.56 (0.50 to 4.61)
Region			0.418
Region A	55.10 (6.70)
Region B	55.27 (5.73)
Region C	56.23 (6.22)

### Survival time

There were 108 (43.0%) recorded deaths during follow-up, with a mean age at death of 59.98 years, SD 5.98, range 46.9– 75.0 years. For men, the mean age at death was 59.99 years (SD 6.88); and for women 59.96 years (SD 4.68). Kaplan-Meier survival time estimates from time of diagnosis revealed a median survival time of 3.78 years (95% CI 3.11– 4.45).

### Predictors of survival

**Fig.2:**
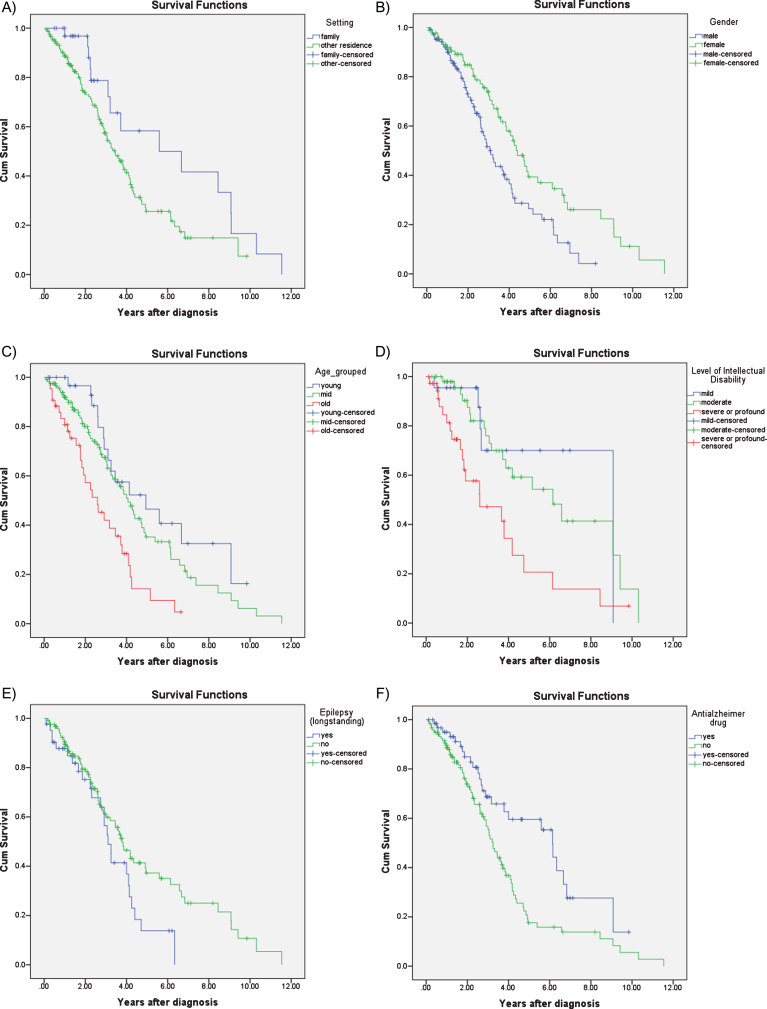
Kaplan-Meier survival curves for A) accommodation setting: family home versus conglomerate setting; B) gender; C) age at diagnosis: young, middle, old; D) level of intellectual disability: mild, moderate, severe; E) epilepsy; and F) treatment with dementia drugs: acetylcholinesterase inhibitors or memantine.

Survival analysis using Kaplan-Meier estimates revealed significant differences between men and women, with shorter survival in men compared to women (median survival in men 3.10 years, 95% CI 2.60– 3.63; women 4.40 years, 95% CI 3.69– 5.11; *p* = 0.004). Kaplan-Meier survival analysis also revealed that age at diagnosis (*n* = 194) was the strongest predictor of survival (*p* < 0.001), such that those diagnosed before age 50 had a median survival of 4.94 years (95% CI 2.22– 7.66), compared to those diagnosed between 50– 60 who had a median survival of 4.06 years (95% CI 3.36– 4.75) and those diagnosed after 60 with a median survival of 2.56 years (95% CI 1.56– 3.58) (Fig. 2C). Level of ID (*n* = 111) was also a significant predictor of survival (*p* = 0.002); median survival was 9.08 years (95% CI not estimated) for mild ID; 6.15 years (95% CI 3.53– 8.78) for moderate ID, and 2.60 years (95% CI 0.44– 4.75) for those with severe ID. The difference between these groups were most noticeable during the first 5 years following diagnosis(Fig. 2D).

Those living with their families also had a survival advantage compared to those that were living in residential settings or independently (median survival 5.59 years, 95% CI 1.13– 10.05 years vs. 3.45, 95% CI 2.80– 4.09; *p* = 0.03) but this difference became less prominent the longer the time lapse after diagnosis (Fig. 2A).

Those with pre-morbid epilepsy (i.e., before the onset of dementia) also had marginally reduced median survival (3.10 years, 95% CI 2.69– 3.50), compared to those without epilepsy (3.78 years, 95% CI 3.06– 4.50; *p* = 0.054). The difference in survival associated with epilepsy was more prominent in the later stages of AD (Fig. 2E). History of depression (*p* = 0.167), sensory impairment (*p* = 0.330), and thyroid dysfunction (*p* = 0.407) were not associated with survival.

Those prescribed anti-dementia drugs had a survival advantage compared to those not prescribed these medications (median survival 6.14 years, 95% CI 5.23– 7.05 versus 3.25 years, 95% CI 2.83– 3.66; *p* = 0.003) (Fig. 2F).

Hazard ratios for these factors adjusted for region are given in Table 3. Multivariate Cox proportional hazards regression was then run including only those variables significantly associated with survival at the 20% level while adjusting for region (*n* = 92). Age at diagnosis (HR 1.07, 95% CI 1.01– 1.14; *p* = 0.021) and moderate ID compared to severe ID (HR 0.42, 95% CI 0.18– 0.98; *p* = 0.045) remained the only significant predictors of survival.

**Table 3:** Estimates of survival based on a Cox regression model – Univariate analyses

	HR+ 95% CI	*p*-value
Age at Diagnosis (*n* = 192)	1.06 (1.03 to 1.09)	<0.001^**^
Sex (*n* = 194)		0.038^*^
Female	reference
Male	1.54 (1.02 to 2.31)
Level of Intellectual Disability (*n* = 110)		0.058
Mild	reference
Moderate	1.13 (0.40 to 3.23)
Severe	2.41 (0.83 to 6.99)
Living (*n* = 184)		0.027^**^
With family	0.51 (0.28 to 0.93)
Other	reference
Epilepsy (*n* = 164)		0.057^*^
No	Reference
Yes	1.59 (0.99 to 2.56)
AD Medication (*n* = 184)		0.015^**^
No	reference
Yes	0.45 (0.24 to 0.86)
History of Depression (*n* = 136)		0.092
No	reference
Yes	0.49 (0.22 to 1.12)
Thyroid Disorder (*n* = 150)		0.617
No	reference
Yes	1.12 (0.71 to 1.77)
Sensory Impairment (*n* = 147)		0.259
No	reference
Yes	1.38 (0.79 to 2.39)

^+^Estimates obtained using Cox regression model adjusted for Site.

A sensitivity analysis was also conducted excluding severity of ID because it had more than 30% missing values. Age at diagnosis (HR 1.04, 95% CI 1.01 to 1.08, *p* = 0.026) and prescription of anti-dementia medication were shown to be significant predictors of survival (HR 0.43, 95% CI 0.21– 0.89; *p* = 0.023) (*n* = 160).

## DISCUSSION

In this large representative clinical cohort of adults with DS and AD the mean age of diagnosis with dementia was 55.8 years of age, and approximately 50% of dementia cases were diagnosed in their 6th decade, with a quarter presenting before age 51, and a further quarter presenting at age 60 or older. Age at diagnosis was associated with gender (men being diagnosed slightly later), living situation, and sensory impairment. Having sensory impairment delayed diagnosis by more than 2 years, and living with family was associated with younger age at diagnosis. Individuals living with family typically were diagnosed more than 4.5 years earlier than those living away from family. However, only living situation remained predictive after adjustment for potential confounders.

Median survival time after diagnosis was 3.78 years, and median age at death was approximately 60 years. Survival time was associated with age of diagnosis, severity of ID, living status, anti-dementia medication status, and history of epilepsy, but none of the other health co-morbidities were predictive of survival. Age at diagnosis and treatment status remained predictive of survival time following adjustment. Similar to findings from the general population, older age of diagnosis was predictive of shorter survival. An increase in age at diagnosis of one year is associated with an increased mortality risk ofapproximately 7%.

### Strengths and limitations

As a consequence of the use of clinical data for the analysis, missing data for some variables was unavoidable. Multivariate analyses were therefore based on a smaller number of cases; however, this remains the largest study of dementia in DS to date. Furthermore, our sampling method ensured a representative sample of individuals with DS with dementia, and captured cases across large regions of Southern England, who had a range of ID severity and comorbidities.

We used age at diagnosis as proxy of age of onset, but this may have underestimated age of onset. Our methodology is, however, comparable to similar studies of AD in the general population, and therefore allows for comparison with these studies. Furthermore, knowledge of typical survival from point of diagnosis is probably more useful for prognostic decisions. Nevertheless, estimates or age of onset and survival may change when there is more emphasis on early diagnosis, particularly if effective treatments become available.

As data were collected from clinical records, and participants were born several decades ago, genetic testing to confirm DS diagnosis was not consistently available. As with any clinical research, it is possible that other unmeasured variables are confounding the relationships found here. Additionally, our health-related measures and medication variables were broadly defined, and clinical assessments were variable between sites, leading to some heterogeneity which may have masked more specific effects. For example, it was not always clear if hearing or visual impairments were corrected. We do not have data regarding reliability across data collection, which may also have contributed toheterogeneity.

### Age of dementia diagnosis

This is the largest cohort of people with DS and dementia to our knowledge, which allows us to provide accurate estimates of the typical age of dementia diagnosis. Our findings demonstrate the young age of dementia diagnosis in the DS population, as well as the considerable variation in age at diagnosis ranging between 35 and 74 years. Typical diagnosis was in the 6th decade, compared to a typical diagnosis age in the 8th-9th decade for SAD. Variation in age at diagnosis may be partially explained by diagnostic issues, such as the difficulty making a diagnosis in the presence of sensory impairments [19]. It is possible that clinicians have misdiagnosed some individuals, though clinical dementia diagnosis in this population has been found to have good inter-rater reliability [20]. Nevertheless, the failure to predict much of the variation in age at diagnosis with most of the variables measured, suggests that unmeasured factors including genetic variability such as *APOE* status also play an important role in individuals with DS. *APOE* genotype has been shown to influence age of dementia onset in the general population, and in the DS population, but genotyping is not currently used in clinical practice [21] and we therefore did not have data on *APOE* status of participants.

In contrast to the general population we did not find a convincing gender difference in age at diagnosis of dementia in the DS population after adjusted analyses, in keeping with other studies [4, 22]. However, one previous study found women to be at higher risk but with no difference in age of onset when *APOE* genotype was accounted for [23] and another found higher risk of dementia in men, along with earlier onset [24].

The brain reserve theory predicts that age of onset of dementia should be earlier in adults with more severe intellectual disability [25] and has been demonstrated in studies of AD in the general population [26]. Baseline severity of ID was not however associated with age of dementia diagnosis in this study of older adults with DS. A few previous studies have demonstrated lower baseline cognitive function to be related to younger age at onset [27], but others have found no association [28]. It is possible that the inherent difficulties in diagnosing dementia in those with more severe disability could mask differences in symptoms at onset, and the amount of missing data for this variable is an additional limitation.

We found a relationship with living situation, which remained significant after adjusting for other variables. The reasons why those living with their families were diagnosed several years before their peers living in other settings are unclear, but the most likely possibility is that family members who know the person with DS well may detect behavior change and other symptoms of dementia at an earlier stage than support staff in communal homes who are more likely to change frequently and therefore know the person less well. It is also possible that those living at home may differ from those living in other settings on other factors, but living situation was not a significant predictor of survival in our adjusted analyses, suggesting that the two groups did not differ on health co-morbidities that are usually associated with poor survival.

### Predictors of survival

In the general population, SAD has a high associated mortality risk, significantly shortening life expectancy, with survival time from disease onset in the general population ranging from 3.3 to 11.7 years across studies [5]. Mortality risk associated with SAD was consistently predicted by age of onset and commonly also by male sex. Furthermore, survival varied from 10.7 years for onset age between 65– 69 years to 4.3 years for onset age between 80– 89 years. However, studies with broader inclusion criteria, i.e., including those with various types of dementia estimated survival to be shorter, while those focused on Alzheimer-type dementia may have longer survival estimates [29]. For example, a recent estimate of survival based on over 400 incident broadly defined dementia cases in a general population cohort found a median survival time post-diagnosis of 4.6 years in women and 4.1 years in men [30].

Survival rates in this study of adults with DS and AD were much shorter if compared against rates from the equivalent age group in the general population; e.g., survival in our study for adults with DS aged 60 and older was between 2 and 3 years, compared to more than 10 years for those of similar age in the general population [30]. Due to the earlier age of dementia onset in the DS population, average age at death was approximately 60 years compared to 90 for women and 87 years for men with dementia in the general population [30]. As in the general population, shorter survival time was strongly predicted by older age of diagnosis.

Other research has also suggested that various health co-morbidities, particularly vascular risks, neuropsychiatric complications [31] as well as dementia severity [32] may be associated with shorter survival time.

This study provides the best estimate of survival in dementia within the DS population to date, and is in keeping with previous estimates from smaller studies in the DS population [33, 34].

Unlike some previous studies which have demonstrated a lack of gender difference in typical life expectancy in the DS population [4], we have shown that in those diagnosed with dementia, men tended to have shorter survival than women in unadjusted though not in adjusted analyses. This may be due to women being diagnosed at a slightly earlier age, suggesting that perhaps signs of AD are more likely to be missed in men, resulting in later age of diagnosis and an apparently shorter survival time.

A few smaller previous studies have suggested associations between the course of AD and DS and baseline cognitive function [35] or epilepsy [36]; however, these findings have not been consistent across studies. We found some association between survival and severity of intellectual disability, with those with more severe disability being associated with worse survival, and also for epilepsy, though this association was no longer apparent after adjustment, suggesting that epilepsy status before the onset of dementia is not a strong predictor of survival. Alternatively, this finding may be explained by documentation of epilepsy at the first assessment reflecting prodromal rather than premorbid epilepsy, with dementia-related epilepsy being diagnosed prior to the dementia.

It was unexpected that those individuals taking any of the current licensed medications for AD would have a significantly longer survival time than those who were not [37], as previous small trials in DS did not show significantly positive effects on cognition. In individuals with SAD, these medications temporarily delay and reduce symptoms, and may delay admission to higher care settings [38], but do not change the course of the disease, though some studies have shown an effect on survival [39]. It is not possible to conclude from the current findings whether improved survival was due to the medications themselves, selection bias, or due to related factors such as improved care and follow-up, or other interventions used in conjunction with medication. Nevertheless, our findings indicate that medication treatment (and associated monitoring and clinical input) is not associated with worse outcomes in DS than in SAD, and thus it is important to highlight equal access to anti-dementia drug treatment in patients with DS and dementia, with a possibility that it may contribute to improved outcomes.

### Implications

The excess of Alzheimer-type dementia in DS is presumed to be largely driven by genetic factors, particularly triplication of the *APP* gene, with AD in DS being a relatively pure form of AD without the general cardiovascular risk factors that is often comorbid with AD in the general population [40]. Our results suggest that there is some validity to this assumption, as we were not able to identify the usual factors associated with age of diagnosis and survival.

Our findings showed that older age/delayed diagnosis of dementia is predictive of shorter survival, and that people living with families were diagnosed earlier than their peers. Some national changes to service delivery for people with ID (including replacing peer-group lifespan day services with individual care providers with more rapid staff turnovers) may act as barriers to the detection of early-onset dementia symptoms in people not living with their families.

Our study demonstrated some role for demographic and health comorbidities as predictors of age at diagnosis, which in combination with genetic factors such as *APOE* status could be used to identify those at most risk for future trials of preventative treatments. The finding that medication such as acetylcholinesterase inhibitors may have a positive impact upon survival in this population is intriguing and deserves further consideration. Important future directions for research could include studies exploring pre-clinical symptoms and mild cognitive impairment and their relationship between age at diagnosis and survival in this population.

This study provides important estimates and insights into possible predictors of survival and age of diagnosis of AD in adults with DS, which will inform selection of participants for treatment trials in the future. In addition, it adds to the literature on clinical presentations associated with genetic forms of AD.
